# Efficient Aggressive Behavior Recognition of Pigs Based on Temporal Shift Module

**DOI:** 10.3390/ani13132078

**Published:** 2023-06-23

**Authors:** Hengyi Ji, Guanghui Teng, Jionghua Yu, Yanbin Wen, Huixiang Deng, Yanrong Zhuang

**Affiliations:** 1 College of Water Resources & Civil Engineering, China Agricultural University, Beijing 100083, China; jihengyi@cau.edu.cn (H.J.);; 2 Key Laboratory of Agricultural Engineering in Structure and Environment, Ministry of Agriculture and Rural Affairs, Beijing 100083, China; 3 Bureau of Agricultural and Rural Affairs, Datong 037000, China

**Keywords:** behavior recognition, pigs, CNN, deep learning, computer vision

## Abstract

**Simple Summary:**

Aggressive behavior can cause severe harm to pigs, especially in poor housing conditions, leading to disease and even death, which causes significant losses for the pig farm. Therefore, the automatic and accurate recognition of aggressive behavior of commercially housed pigs is important for pig farm production management. In this study, we proposed a video behavior recognition method based on the temporal shift module (TSM) that can detect whether aggressive behavior occurred in pig groups automatically. TSM is a convolutional neural network module to process video sequence data. Experimental results demonstrated that the method can recognize pig aggression effectively, which helps improve the use of automated management techniques in pig farming.

**Abstract:**

Aggressive behavior among pigs is a significant social issue that has severe repercussions on both the profitability and welfare of pig farms. Due to the complexity of aggression, recognizing it requires the consideration of both spatial and temporal features. To address this problem, we proposed an efficient method that utilizes the temporal shift module (TSM) for automatic recognition of pig aggression. In general, TSM is inserted into four 2D convolutional neural network models, including ResNet50, ResNeXt50, DenseNet201, and ConvNext-t, enabling the models to process both spatial and temporal features without increasing the model parameters and computational complexity. The proposed method was evaluated on the dataset established in this study, and the results indicate that the ResNeXt50-T (TSM inserted into ResNeXt50) model achieved the best balance between recognition accuracy and model parameters. On the test set, the ResNeXt50-T model achieved accuracy, recall, precision, F1 score, speed, and model parameters of 95.69%, 95.25%, 96.07%, 95.65%, 29 ms, and 22.98 M, respectively. These results show that the proposed method can effectively improve the accuracy of recognizing pig aggressive behavior and provide a reference for behavior recognition in actual scenarios of smart livestock farming.

## 1. Introduction

The modern pig farming industry faces the challenge of aggressive behavior among pigs while pursuing efficiency and scale [1]. Aggressive behavior became a common phenomenon due to the mixing of pigs in group management [2]. This behavior not only causes physical harm to pigs, but also impacts growth, reproductive rate, welfare, and may even lead to disease and death, resulting in economic losses for pig farms [3,4]. The development of automatic detection and recognition technology for aggressive behavior became crucial in improving pig health and production efficiency [5]. The computer vision-based automatic recognition of aggressive behavior enables real-time monitoring and early warning of aggressive behavior by analyzing and recognizing behavior characteristics [6]. This technology has the potential to increase monitoring accuracy and efficiency, while also decreasing labor costs by avoiding the issue of missed detection during manual observation [7,8]. Therefore, the development of automatic recognition technology for aggressive behavior is of great significance for improving pig welfare and economic benefits.

The typical aggressive behaviors among pigs involve ear biting, tail biting, chasing, and trampling, which constitute a fast, complex, and dynamic form of interaction [9]. These actions possess multiple spatial features such as pig location, speed, and angle with continuous dynamic change. Therefore, achieving recognition of aggressive behavior requires the simultaneous consideration of both spatial and temporal features. Some scholars recognized aggressive behavior by traditional machine learning techniques. Viazzi et al. [10] used motion history images to extract information on pig movement, including mean intensity and occupancy index, and applied linear discriminant analysis to recognize aggressive behavior. Oczak et al. [11] developed an automated detection method for pig aggression using the activity index of the pig group and a multi-layer feedforward neural network. Lee et al. [12] used a Kinect depth sensor to extract activity features in the pig pen and applied two binary support vector machines in a hierarchical manner for aggressive behavior recognition. Chen et al. [13] developed an energy model to identify pig aggression by using aggressive key-frame sequences and kinetic energy differences between adjacent frames extracted from video data. Liu et al. [14] extracted the activity index of fattening pigs in a dynamic background environment using an adaptive learning rate Gaussian mixture model and applied a support vector machine classifier for aggressive behavior recognition. The above methods utilized image processing techniques to extract handcrafted features, and they then used traditional machine learning models to recognize aggressive behavior in pigs. However, this strategy was restricted by high workload low efficiency and accuracy due to pig adhesion, occlusion, lighting, growth, and behavioral patterns. Furthermore, it is highly sensitive to factors such as image quality and pig-body contact, reducing the stability and robustness of the model.

Compared to traditional machine learning methods, deep learning is capable of more accurate recognition of aggressive behavior by learning more complex and advanced feature representations automatically [15]. Moreover, it can process and learn temporal features of videos, better capturing the dynamic changes in aggressive behavior [16]. Chen et al. [17] utilized VGG-16 to extract spatial features and then fed these features into long short-term memory (LSTM) for further extraction of temporal features, followed by processing video segments directly to identify pig aggression. Liu et al. [18] simplified group behavior into pairwise interactions using a tracking-detection algorithm, and they then combined convolutional neural networks (CNN) and recurrent neural networks (RNN) to extract both spatial and temporal features for tail biting behavior recognition. Hakansson et al. [19] used CNN to extract spatial information and integrated two secondary networks (LSTM and CNN) to develop a computer vision-based method for detecting tail-biting in pigs. Gao et al. [20] proposed a hybrid model combining CNN and gated recurrent units and used spatio-temporal attention mechanisms to recognize aggressive behavior of pigs automatically. The aforementioned methods were all based on hybrid models, where spatial features were initially extracted using CNN, and these features were then input into LSTM or RNN for extracting temporal features. However, these methods sacrificed the low-level temporal modeling ability to improve efficiency, resulting in the loss of crucial information during feature extraction before temporal fusion.

In summary, recognizing aggressive behavior of pigs requires an efficient method and does not rely on hybrid models. The temporal shift module (TSM) was developed to address this. TSM is a convolutional neural network module to process video sequence data, which enhances the model’s nonlinear feature extraction ability by shifting the input feature in the time dimension [21]. This module can be inserted in existing 2D CNNs to achieve time modeling of zero computation and zero parameters. Although TSM was widely used in tasks such as video classification and action recognition [22,23], no researcher applied TSM to the field of pig farming. In this study, we aimed to insert TSM into four widely used 2D CNN models, which enhance the model’s learning ability on time features, while maintaining the model’s performance in handling spatial features. The modified models were then used for the automatic recognition of aggressive behavior.

This paper comprises four primary sections. The Section 2 provides an overview of the current status of pig aggression recognition and highlights the advantages of using TSM. The Section 3 provides a detailed descriptions of data sources, data collection methods, criteria for dataset construction, TSM and its utilization, model hyperparameters, and evaluation metrics. The Section 4 presents the performance of the TSM-based model in pig aggression recognition and compares it with other current behavior recognition models. Additionally, the visualization of feature maps generated by the model was conducted to analyze how the model recognized pig aggression behavior and the reasons behind misclassifications. The Section 5 elucidates the conclusions drawn from this research and offers future prospects.

## 2. Materials and Methods

### 2.1. Data Collection

The experiment was conducted at the Research Base for Pig Nutrition and Environmental Control in Rongchang District, Chongqing City. The study area consisted of 50 pig pens of 4.2 m × 2.5 m containing one feeding trough and four drinking fountains. The flooring was composed of semi-slat floors with a gap of 1.2 m. Two toys made of iron chains (75 cm) and PVC materials were placed in each pen for the pigs to play with. Due to the frequent aggressive behavior exhibited by pigs when mixed together to establish a hierarchy, two pens of mixed pigs were selected for one week of video recording in August 2022. The initial average weight of the pigs before recording was 40 kg, and their average weight after recording was 44.5 kg. The pigs were of the Duroc × Landrace × Yorkshire breed, with eight pigs in each pen. The feed was replenished twice daily at 8:00 a.m. and 2:00 p.m., and the pens were cleaned and disinfected at 3:00 p.m. The artificial lighting in the pen was turned on from 7:00 a.m. to 8:00 p.m. daily. 

A high-definition 2D camera (Hikvision DS-2CD3326DWD-I network camera, 1920 × 1080 P, 25 frames per second, Hangzhou Hikvision Digital Technology Co., Ltd., HaiKang, Shenzhen, China) was installed on the top of the pigpen, which was connected to a hard disk recorder with a 3TB hard drive to achieve vertical overhead recording. The video was recorded from 8:00 a.m. to 6:00 p.m. daily. 

### 2.2. Dataset

The obtained videos need to be annotated manually to establish a dataset containing videos of both aggressive and non-aggressive behaviors. Referring to the definitions of aggressive and non-aggressive behaviors in other literature [20], the definitions of the behaviors collected in this study are shown in Table 1. The collection process was not interfered with human intervention. The videos were observed by professionals to distinguish aggressive and non-aggressive behaviors accurately. A total of 1 h and 27 min of edited aggressive behavior videos and an equal amount of non-aggressive behavior videos were obtained. 

**Figure 1:**
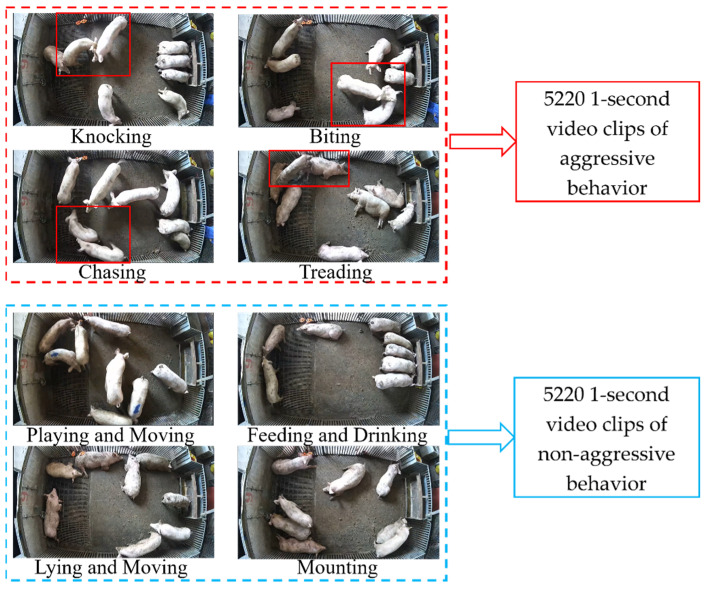
Composition and examples of aggressive and non-aggressive behaviors in datasets.

Due to the fact that the pig group exhibiting aggressive behavior was a mixed group, the duration of the aggressions was long and the frequency was short. After observing the videos and considering the processing methods for the duration of aggressive behavior videos in other literature [18], the videos with different durations were edited to a uniform length of 1 s to expand the dataset. Therefore, the dataset consisted of 5220 1 s videos of aggressive behavior and 5220 1 s videos of non-aggressive behavior, totaling 10,440 videos. In order to reduce training time due to hardware limitations, all videos were scaled to 720 × 480 P. Examples and composition of the dataset are shown in Figure 1. Since multiple behaviors tended to occur simultaneously, only the main behaviors of the pigs in the figure are described. The dataset was divided into a training set, a validation set, and a test set at a ratio of 6:2:2, with 6264 videos used for training, 2088 videos for validation, and 2088 videos for testing. 

### 2.3. Temporal Shift Module

**Figure 2:**
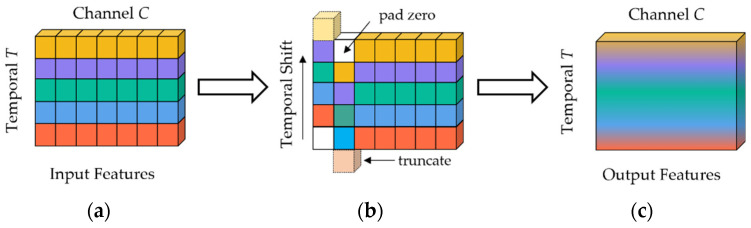
Schematic diagram of temporal shift module: (**a**) the input features with C channels and T frames; (**b**) TSM moving the features along the temporal dimension; (**c**) the output features mingles both past and future frames with the current frame.

As pig aggression is a complex interactive and rapidly occurring behavior, the model with ability to learn spatio-temporal features was required for recognition. TSM is an efficient cross-frame processing module that enhances the feature extraction ability of 2D CNN for video data, and it improves the accuracy of video classification. It is also a zero-parameter module that does not increase additional computational complexity. The schematic of TSM is shown in Figure 2. The input feature is a tensor with C channels and T frames, with different time-stamped features represented in different colors in each row. TSM shifts a portion of the channels one step forward along the time dimension and a portion of the channels one step backward along the time dimension, with the empty space filled with zeros. By shifting a portion of the channel information in the input feature at each time stamp, TSM can fuse the spatial semantic information of adjacent frames into the current frame and promote temporal information interaction between adjacent frames. To provide a more detailed explanation of TSM, we use the example of one-dimensional convolution with a kernel size of 3. Let W = (w_1_, w_2_, w_3_) be the weights of the convolution, and X be an infinitely long one-dimensional vector. The convolution operator Y = Conv(W, X) can be expressed as: Y_i_ = w_1_X_i−1_ + w_2_X_i_ + w_3_X_i+1_. TSM decomposes the convolution operation by two steps: shifting and multiplication-accumulation. Specifically, TSM shifts the input vector X by −1, 0, and +1, and multiplies each shifted version by w_1_, w_2_, w_3_, respectively, and then accumulates them to obtain Y, as shown in Equations (1) and (2):

(1) $$X_{i}{}^{-1}=X_{i-1},\;X_{i}{}^{0}=X_{i},\;X_{i}{}^{+1}=X_{i+1}$$

(2) $$Y=w_{1}X^{-1}+w_{2}X^{0}+w_{3}X^{+1}$$

The first step of shifting can be performed without any multiplication; thus, it does not add any computational cost. Although the second step has a relatively higher computational cost, TSM combines multiplication and accumulation into the subsequent 2D convolution. Compared to 2D CNN models, TSM does not introduce additional computational cost. Additionally, since the channel information flows bidirectionally along the time dimension, the information from adjacent frames is merged with the current frame after the shift operation. When a 2D convolution is applied to the output of TSM, it is equivalent to performing a 3D convolution between adjacent frames. It compensates partially for the dynamic feature extraction capability that is lacking in 2D convolutions. Therefore, TSM can be inserted into any existing 2D CNNs, enabling it to have the performance of a 3D CNNs while maintaining the computational cost of 2D CNNs.

It should be noted that if a large number of channels are shifted, it may lead to an increase in memory overhead and a weakening of the model’s ability to model spatial features, ultimately resulting in a decrease in performance. Therefore, in this study, we only moved a small number of channels to model temporal dynamics, setting the proportion of bi-directional channel shifts to only 1/4. 

### 2.4. Different CNN Models with Temporal Shift Module

Although TSM is a module designed to facilitate the learning of temporal features, the excessive channel shifting can lead to a decline in a model’s ability to learn spatial features. Therefore, it is necessary to balance the temporal and spatial feature learning capabilities of model. In this study, four high-performing models were selected to insert TSM for the recognition of pig aggressive behaviors, including ResNet50 [24], ResNeXt50 [25], DenseNet201 [26], and ConvNext-t [27]. These four models were all built based on blocks with residual connections. TSM was inserted before the first convolutional layer in each block, so the shifting operation only occurred in the residual mapping branch. Due to the presence of the identity mapping structure in the residual network, the original spatial semantic information can still be transmitted to subsequent network layers completely and preserving the model’s spatial feature learning ability effectively. No adjustments were made to the four models except for TSM insertion.

**Figure 3:**
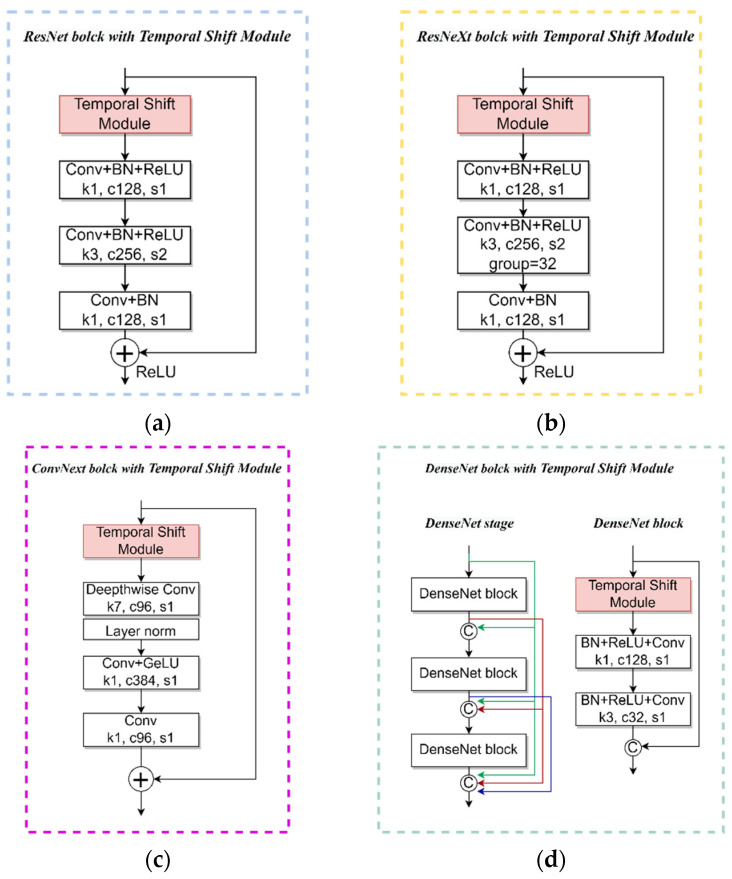
Schematic diagram of inserting TSM in blocks of different 2D CNN models: (**a**) ResNet block with temporal shift module; (**b**) ResNeXt block with temporal shift module; (**c**) ConvNext block with temporal shift module; (**d**) DenseNet block with temporal shift module and dense connections in the DenseNet stage.

ResNet50 is a deep CNN model composed of ResNet blocks. The core idea of a ResNet block is to add the input and output to form a skip connection, thereby alleviating the problems of gradient vanishing and network degradation. ResNet50 consists of 5 stages; the first stage contains 1 convolutional layer and 1 max pooling layer. Each of the following 4 stages contains 3, 4, 6, 3 ResNet blocks, respectively, and each ResNet block is composed as shown in Figure 3a. After completing the feature extraction from the five stages, a global average pooling layer and a fully connected layer were applied to output the classification results.

ResNeXt50 is an improved model based on ResNet50. It is characterized by the use of group convolution in each block, which splits the 3 × 3 convolutional layers in each ResNet block into multiple parallel sub-convolutional layers. The composition of ResNeXt block is shown in Figure 3b. This group convolution technique not only reduces the model parameters, but also improves the accuracy and generalization ability of the model effectively. The structure of ResNeXt50 is similar to ResNet50, consisting of 5 stages, but the stages are designed based on ResNeXt blocks.

ConvNext-t is also an improved model based on ResNet50. The composition of its block is shown in Figure 3c. It is featured by replacing the 3 × 3 convolutional layer in the block with a 7 × 7 depth-wise convolution and moving it forward to the first convolutional layer; using layer normalization instead of batch normalization, and only using it after the first convolutional layer; replacing the ReLU activation function with GeLU and only using it in the second convolutional layer. The structure of ConvNext-tiny is also similar to ResNet50, consisting of 5 stages. The first stage contains 1 convolutional layer and 1 layer normalization, and each of the following four stages contains 3, 3, 9, and 3 ConvNext blocks. After feature extraction from the five stages, the model outputs the classification results through a global average pooling layer, a layer normalization layer, and a fully connected layer.

DenseNet-201 is a dense connected CNN model, which is characterized by each layer connected to all previous layers, thereby enhancing feature propagation and reuse, reducing the number of parameters and computational complexity. DenseNet-121 consists of 4 stages and 3 transition layers, as shown in Figure 3d. In each block of the stage, the dimension is reduced by a 1 × 1 convolution, features are then extracted by a 3 × 3 convolution, and finally, the outputs of all layers are concatenated in the channel dimension. Each transition layer consists of a 1 × 1 convolution and an average pooling layer, which is used to reduce the size and channel number of the feature maps. It is notable that only three blocks are shown to illustrate the dense connection of the DenseNet stage due to the limitation of the picture, in Figure 3d. In fact, DenseNet-121 contains 6, 12, 48, and 32 blocks, respectively. In the last stage, there is a global average pooling layer and a fully connected layer that outputs the model’s classification results.

### 2.5. Model Training Parameters

The experimental platform used in this study consisted of an 8-core 32GB CPU and a Tesla T4 16 GB GPU. The PyTorch 1.8.1 deep learning framework was built on Python 3.8 and CUDA-Toolkit 10.2 for both model training and testing. During training, the stochastic gradient descent algorithm with momentum was used for model parameter tuning. The batch size was set to 8 and a total of 60 epochs were trained. The initial learning rate was set to 0.00125, the momentum was set to 0.9, and the weight decay coefficient was set to 0.0001. To prevent gradient explosion, gradient clipping was employed with a maximum norm of 40 and L2 norm was used for clipping. All models were learned based on transfer learning initialized by pre-trained model weights on ImageNet-1K and fine-tuned.

### 2.6. Evaluation Metrics

To evaluate the performance of the proposed model, we used accuracy, recall, precision, F1-score, speed, and model parameters as evaluation metrics to verify the effectiveness of our method. The formulas for these metrics are as follows:

(3) $$\mathrm{Accuracy}=\frac{\mathrm{TP}+\mathrm{TN}}{\mathrm{TP}+\mathrm{FP}+\mathrm{TN}+\mathrm{FN}}$$

(4) $$\mathrm{Recall}=\frac{\mathrm{TP}}{\mathrm{TP}+\mathrm{FN}}$$

(5) $$\mathrm{Precision}=\frac{\mathrm{TP}}{\mathrm{TP}+\mathrm{FP}}$$

(6) $$F1=2\times \frac{\mathrm{Recall}\times \mathrm{Precision}}{\mathrm{Recall}+\mathrm{Precision}}$$

In the above formula, TP represents the number of videos of pig aggressive behavior recognized correctly, FP represents the number of videos of non-aggressive pig behavior recognized incorrectly as aggressive, TN represents the number of videos of non-aggressive pig behavior that are correctly recognized, and FN represents the number of videos of aggressive pig behavior recognized incorrectly as non-aggressive.

Speed refers to the time cost to process a video. The real-time performance of the model improves with a shorter average process time.

Model parameters refer to the number of parameters learned and adjusted during the training process, which is usually considered as a metric for measuring model complexity.

## 3. Results

### 3.1. Evaluate the Performance of the Four 2D CNN Models with Temporal Shift Module

**Figure 4:**
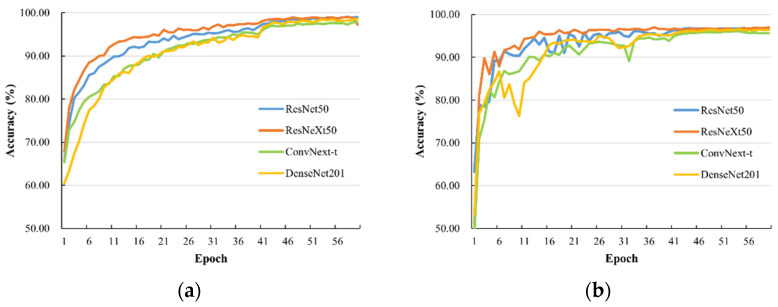
The accuracy of ResNet50, ResNeXt50, ConvNext-t, and DenseNet201 inserted with TSM: (**a**) accuracy on the training set, (**b**) accuracy on the validation set.

Figure 4 shows the accuracy of four CNN models with TSM inserted during the training process on the training and validation sets. As shown in Figure 4a, all four models exhibited a relatively smooth optimization trend on the training set, with ResNeXt50 showing slightly better convergence speed than the other three models. The highest accuracy rates achieved by ResNet50, ResNeXt50, ConvNext-t, and DenseNet201 on the training set were 99.05%, 99.04%, 97.91%, and 98.57%, respectively. As shown in Figure 4b, ResNeXt50 tended to converge after 26 epochs on the validation set, while the other three models required 41 iterations to converge. The highest accuracy rates achieved by ResNet50, ResNeXt50, ConvNext-tiny, and DenseNet201 on the validation set were 96.73%, 97.01%, 96.06%, and 96.49%, respectively.

These results indicate that the inclusion of TSM improved the performance of the four CNN models significantly in recognizing pig aggressive behavior. Notably, ResNeXt50 demonstrated the highest accuracy on the validation set, thereby corroborating its performance on the training set. This could be attributed to the employment of group convolution structure in ResNeXt50, which enhanced the model’s ability to acquire features in the data at an accelerated pace, facilitating rapid adaptation to the training data and convergence in fewer iterations.

Table 2 presents the recognition results of the four models inserted with TSM on the test set. The results show that ResNeXt50 achieved the highest accuracy, precision, and F1 score, consistent with its outstanding performance on the training and validation sets. On the other hand, ResNet50 showed the best performance in recall rate, with a relative increase of 0.45%, 1.5%, and 0.36% compared to ResNeXt50, ConvNext-tiny, and DenseNet201, respectively. It was suggested that ResNeXt50 outperformed the other three models in accurately classifying non-aggressive behavior videos. This could be attributed that the group convolution in ResNeXt50 can capture spatio-temporal features when processing video data, thereby improving the accuracy and robustness of the model. In terms of model parameters, ResNeXt50 had fewer parameters than ResNet50 and ConvNext-tiny, and only slightly more parameters than DenseNet201 by 4.88 M. This indicates that ResNeXt50 can still maintain high accuracy while reducing the number of model parameters.

In conclusion, ResNeXt50 was the best-performing model among the four models considering the performance of ResNeXt50 on the training, validation, and test sets, as well as its parameter count and computational resource consumption. Therefore, ResNeXt50 will be used as the baseline model for the subsequent experiments, and the ResNeXt50 model inserted with TSM will be referred to as ResNeXt50-T.

### 3.2. Visual Analysis of Temporal Shift Module

**Figure 5:**
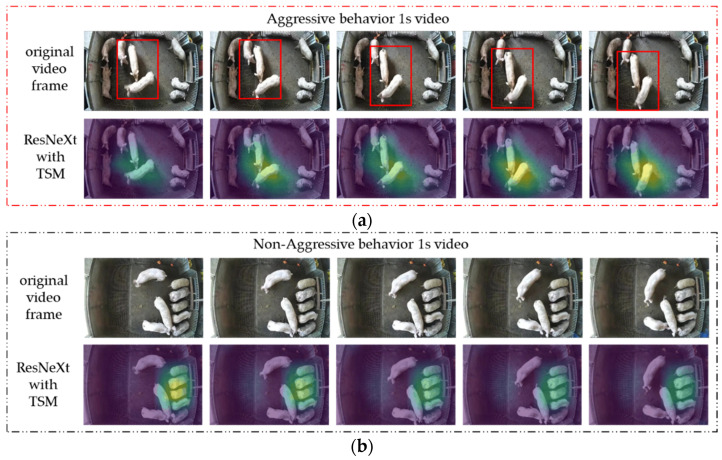
ResNeXt50-T heat map comparison after extracting video features of aggressive and non-aggressive behaviors: (**a**) when the two pigs located in the center of the video engaged in mutual biting with significant displacement, ResNeXt50-T successfully focused on their positions; (**b**) when no aggressive behavior was observed in the video, ResNeXt50-T exhibited a tendency to focus on the region where the pigs are clustered.

Although deep learning models have poor interpretability, Grad-CAM [28] can analyze the attention areas of a deep learning model for a specific class and present them in the form of heat maps overlaid on the original image. It could be adopted to analyze whether the model learned the correct features or information by examining the areas of interest. To better understand how ResNeXt50-T distinguishes between pig aggressive and non-aggressive behavior, we used Grad-CAM to visualize the features extracted by ResNeXt50-T as heat maps and analyzed them. Feature extraction in ResNeXt50-T is mainly performed in five stages, and so, we chose to visualize the output features of the last ReLU activation function in the third block of the fifth stage. Figure 5 shows a comparison of the heat maps of aggressive and non-aggressive behavior after ResNeXt50-T extracted the video features. Since the original 1 s video contained 25 frames, we selected every fifth frame to display the original video frame and its corresponding heat map in order to show the behavior changes.

When aggressive behavior occurred, it can be observed that one pig was biting another pig’s body with obvious displacement, while the other pigs were almost motionless. Consequently, the temporal and spatial information in the video was mainly generated by the pig’s aggressive behavior. In this case, ResNeXt50-T recognized the pig’s aggressive behavior successfully as shown from the heatmap. In particular, the highlighted area in the heat map corresponded to the part of the pig’s body where the aggression occurred, which further demonstrated ResNeXt50-T’s ability to extract features related to pig aggression effectively.

In the video without any aggressive behaviors, three pigs were observed eating while two pigs stood beside the feeding trough without any movement. Two pigs were in light contact with their heads, while one pig was walking without any contact with other pigs. Under this condition, multiple types of behaviors were often observed simultaneously, and spatial-temporal information was distributed sporadically throughout the video. It was complex to extract spatio-temporal features, since there was no requirement to recognize a specific type of behavior in the model. From the generated heat map, it was observed that ResNeXt50-T mainly focused on the three pigs that were eating, while neglecting the single pig that caused significant displacement or the two pigs in contact. This suggests that ResNeXt50-T may judge the presence of aggressive behavior based on significant displacement caused by interactions among two or more pigs. In the absence of such characteristics, attention was directed towards the area where most pigs gathered. This also demonstrates that the temporal features of aggressive behavior is a critical factor in differentiating it from non-aggressive behavior, and ResNeXt50-T can extract the temporal features of aggressive behavior effectively.

### 3.3. Comparison of Aggressive Behavior Recognition Results of Different Classification Models

ResNeXt50-T was compared to C3D + Linear Classifier, CNN+LSTM, TSN, R2plus1D, and I3D on the dataset through comparative experiments. Among which, C3D + Linear Classifier, R2plus1D, and I3D are 3D CNN models based on 3D convolution. CNN + LSTM is a widely used method for recognizing aggressive behavior in pigs. TSN is also a model that performs temporal modeling on 2D CNN model to enable it to capture spatio-temporal information.

Table 3 presents the comparison results among six classification models. ResNeXt50-T outperformed the other models with higher accuracy and F1 scores. Compared to C3D + Linear Classifier, ResNeXt50-T achieved an accuracy and F1 score improvement of 6.77% and 6.83%, respectively. It could be explained that TSM enabled the model to perform temporal modeling and extract the temporal features of aggressive behavior effectively. For the purpose of recognizing aggressive behaviors in pigs in intensive pig farming, our objective was to achieve real-time monitoring within the facility. The cost of deploying the algorithm is a crucial factor that must be considered. We evaluated two critical metrics: model size and inference speed. The use of excessively large models can present challenges in terms of loading and running, increasing the hardware requirements. Hence, it is essential to select models that are appropriately sized for efficient deployment and execution on cost-effective devices. ResNeXt50-T only involves parameters of ResNeXt50 itself because TSM requires no additional parameters and computation. Moreover, the group convolution in ResNeXt50 reduces the parameter count, and so, ResNeXt50-T had the smallest model parameters. Compared to the widely used CNN + LSTM in pig aggressive behavior recognition, ResNeXt50-T achieved an accuracy and F1 score improvement of 4.17% and 4.16%, respectively, and reduced the model parameters by 1.32 M. The proposed method in this paper had relatively lower hardware requirements compared to other models, making it more advantageous for deployment on mobile devices or edge devices at a lower cost. In real-time monitoring scenarios, it is crucial to ensure fast detection and recognition capabilities along with instantaneous response times. Considering the hardware limitations, the speed of different models was evaluated on the GTX 1660ti GPU. The results demonstrated that both ResNeXt50-T and CNN + LSTM achieved the fastest detection speed for individual videos, both at 29 ms. However, ResNeXt50-T showcased higher detection accuracy, thus better meeting the demands for real-time detection of aggressive behaviors in intensive group pig farming.

In summary, the proposed method can improve the recognition accuracy of aggressive behavior and provide a new approach for pig behavior recognition with high efficiency.

### 3.4. Visual Analysis of Model Misclassification Results

**Figure 6:**
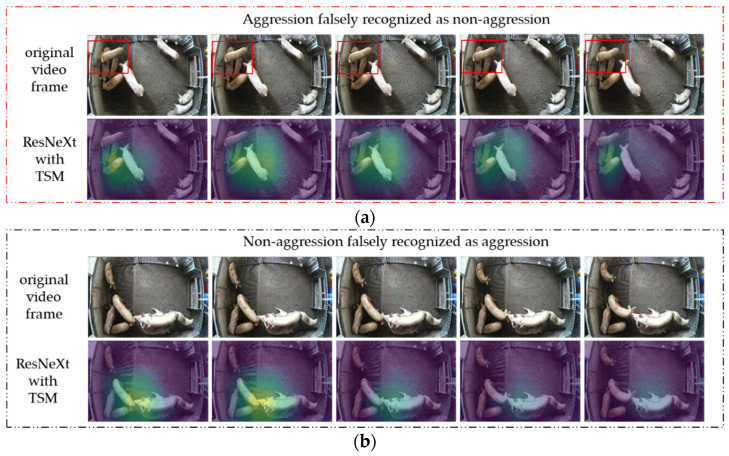
Examples and heat maps of video misclassification by ResNeXt50-T: (**a**) due to two pigs in the top left corner exhibited treading behavior, characterized by a significant overlap of the pigs’ bodies without notable displacement, ResNeXt50-T encountered difficulties in recognizing aggressive behavior; (**b**) due to the overlapping of the heads of the three pigs in the middle with significant displacement, ResNeXt50-T erroneously recognized this behavior as an aggression.

Figure 6 presents two examples of misclassifications made by ResNeXt50-T. To further understand the reasons for the misclassifications, we also visualized these examples as heatmaps using Grad-CAM, with every fifth frame selected for visualization. When the aggressive behavior video was misclassified as a non-aggressive behavior video, the pig was observed treading on top of another pig’s body in the top left corner of the video. However, ResNeXt50-T failed to recognize this as an aggressive behavior. The heatmap revealed that ResNeXt50-T’s focus was not on the pig involved in the aggressive behavior, but on the four pigs in close proximity. There were two reasons for this misclassification. Firstly, the pig being trod on did not make an obvious physical response to the aggression yet, so there were not enough distinct spatio-temporal features for the model to extract. Secondly, there was a large overlap between the pigs’ bodies, suggesting a tendency for mounting behavior, which was difficult for ResNeXt50-T to recognize aggressive behavior. This heat map was consistent with the findings in Section 3.2 that ResNeXt50-T tended to focus on clustered areas among pigs when it failed to detect aggressive behavior.

In the example of non-aggressive behavior video being misclassified as aggressive behavior video, two pigs were seen performing a mounting behavior on the bottom of the video, and one pig’s head was near the heads of the pigs performing the mounting behavior. However, there was no aggressive behavior among the three pigs. ResNeXt50-T’s attention was focused on the heads of the three clustered pigs from the heat map, potentially due to the misinterpretation of the ear biting or head butting behaviors. The misrecognition as an aggressive behavior could be attributed to the misleading spatiotemporal information of the overlap between the pig heads and the slight head movement of the left pig. Combining the two examples, we conclude that ResNeXt50-T was more prone to misrecognition behaviors with overlapping features, ambiguous behavior, and unclear temporal information.

## 4. Discussion

In this study, we inserted TSM into four mainstream 2D CNN models to recognize pig aggressive behaviors. The proposed method improved the recognition accuracy of aggressive behaviors without increasing any additional model parameters. Compared to traditional computer vision and machine learning methods [10,11,12,13,14], the proposed method realized an end-to-end aggressive behavior recognition model without manual design of spatio-temporal features, which were all completed by the model itself. Previous CNN + LSTM method adopting deep learning sacrificed the temporal modeling ability of the low-level layers of the model to improve efficiency and loss some key information [17,18,19]. Our proposed method based on TSM is capable of both high efficiency and high performance. According to the results in Table 3, the proposed method had higher accuracy and fewer model parameters. Gao et al. [20] used the CNN + GRU method with spatio-temporal attention mechanism to recognize aggressive behavior. However, they used VGG16 as the backbone, which had 138 M model parameters, the model parameters of this method were relatively large, limiting its practical application value in pig farms. In contrast, our proposed ResNeXt50-T consisted of 22.98 M model parameters, making it easier to apply in practical pig farming. Another characteristic of the proposed method was that TSM had better adaptability. TSM can be inserted into any existing 2D CNN model, not only to transform 2D CNN models into models with spatio-temporal modeling ability, but also to be inserted into existing behavior recognition models to improve recognition accuracy.

Another contribution of this study was the use of Grad-CAM to visualize the features extracted by ResNeXt50-T as heat maps. It facilitates to analyze whether ResNeXt50-T learned useful information directly. In the CNN + LSTM method for recognizing aggressive behavior, visualizing the features extracted by CNN only revealed changes in spatial features without providing insights into how LSTM processes temporal features. Our method was prone to be understood and accepted, because the heat map visualizing the features extracted by ResNeXt50-T represented the learning information during training. By combining Figure 5 and Figure 6, it concludes that ResNeXt50-T recognizes aggressive behavior by accurately focusing on the pig involved in the aggression. However, a common limitation of deep learning methods is the lack of interpretability. For ResNeXt50-T, it is still unclear how it made judgments on more complex non-aggressive behavior videos. Currently, the model tended to focus on clustered pigs in non-aggressive behavior videos. In future research, we will consider analyzing how the model recognizes non-aggressive behavior and, thus, establish a more suitable dataset to improve recognition accuracy.

The pigs involved in this research were in the fattening stage and were maintained at a stocking density of 1.3 m^2^/pig. Previous studies, such as Fu et al., [29] suggested that a stocking density of 1.2 m^2^/pig is suitable for fattening pigs, as it promotes increased positive social behavior during pig mixing. The stocking density in our study was slightly below this recommended threshold, indicating that the pigs were not subjected to a relatively crowded environment. Therefore, we assert that the stocking density used in our experimental setup did not contribute to an elevated occurrence of aggressive behavior among the pigs. Nevertheless, it is worth noting that a significant proportion of recorded aggressive behaviors did occur during pig mixing. Consequently, we propose that further reductions in stocking density during pig mixing can potentially diminish the incidence of aggressive behavior. Traditional manual observations of pig behavior are often limited by human constraints, rendering a comprehensive assessment of pig behavior challenging. In contrast, the method proposed in our study enables continuous 24 h monitoring of pig groups to ascertain aggressive behaviors. This approach allows for the recording of the frequency and duration of aggressive behaviors, facilitating a more objective evaluation of pig welfare within the pigsty.

Although our method achieved recognition of pig aggressive behavior efficiently, there are still some limitations to be addressed. The dataset used in this study had a video duration of 1 s, while previous studies used datasets with video durations of 1, 2, and 3 s, indicating a lack of standardization in the dataset creation process. For future investigations, it is advisable to generate datasets with varying durations by utilizing the same set of data. This approach enables an in-depth analysis of a model’s performance on datasets with different durations. Furthermore, it is recommended to evaluate models trained on a specific duration by testing them on datasets of varying durations. By comprehensively considering the performance of models under both conditions, it became possible to determine the optimal standard for video duration within the dataset. This study demonstrated that inserting TSM into a 2D CNN model can achieve recognition of aggressive behavior. It is noteworthy that the adopted 2D CNN model was the original unmodified model. Many studies improved CNN model structures to suit their application scenarios in pig behavior recognition research [30,31]. The further improved accuracy is expectable if appropriate optimizations are made to the 2D CNN model for aggressive behavior recognition. The model proposed in this paper was specifically designed and applied for the recognition of attack behaviors. However, it should be emphasized that the model possessed the ability to process spatio-temporal features in videos. Consequently, it holds promise for recognizing a wide range of positive behaviors, including nursing, feeding, and toy playing, among others. Expanding the application of the proposed method to recognize and evaluate such positive behaviors would provide valuable insights into assessing the welfare status of pigs.

## 5. Conclusions

In this paper, we proposed a pig aggressive behavior recognition method based on TSM. The method utilized aggressive behavior videos and non-aggressive behavior videos as inputs and inserted TSM into ResNet50, ResNeXt50, ConvNext-t, and DenseNet201 to improve these four 2D CNN models’ ability to process spatio-temporal features. By testing the four improved models on our dataset, the ResNeXt50-T model possessed the best balance with accuracy, recall, precision, F1 score, speed, and model parameters on the testing set of 95.69%, 95.25%, 96.07%, 95.65%, 29 ms, and 22.98 M, respectively. To further understand TSM’s contribution to the model’s temporal modeling, we used GradCAM to visualize the aggressive and non-aggressive behavior features processed by ResNeXt50-T as heat maps. The results showed that ResNeXt50-T can determine whether the aggressive behavior occurred in the video by focusing on the pig of aggression accurately. However, the specific mechanism by which the model discriminated non-aggressive behaviors remained unclear, as the model’s attention predominantly focused on clustered pigs, based on our analysis. Future research efforts should be dedicated to exploring this aspect in more detail. In the existing studies on pig aggressive behavior recognition, the datasets exhibited variations in terms of video durations. Hence, it is recommended to establish standardized criteria for dataset creation in future endeavors. In this study, the 2D CNN model with TSM inserting was utilized without any specific improvements. However, it is expected that incorporating appropriate optimizations tailored to the specific application scenario would yield better performance. Furthermore, the method proposed in this paper can be extended to address other behavior recognition tasks involving spatio-temporal features, thereby offering a promising solution for future advancements in pig behavior analysis.

## Figures and Tables

**Table 1:** An ethogram of pig aggressive and non-aggressive behaviors.

Classification	Type	Description
Aggression	Biting	Pigs open its mouth, make contact with another pig’s ear, tail or body, and then closes its mouth.
	Knocking	Pigs use its head to swiftly push and jostle the head or body of other pigs.
	Treading	Pigs forcefully stand on the body of other pigs with its feet.
	Chasing	Pigs intentionally continue to pursue and attack other pigs.
Non-aggression	Feeding	Pigs insert their heads into the feeding trough.
	Drinking	Pigs biting the drinking fountain.
	Moving	The slow alternating movement of the limbs propels the body forward without any other behavioral expression.
	Playing	Sniffing, biting or nuzzling the pig toys and nuzzling some part of another pig.
	Mounting	Pigs approach motionless pig and placing its foreleg span on it with a long time.

**Table 2:** Recognition results of ResNet50, ResNeXt50, ConvNext tiny, and DenseNet201 inserted with TSM on the test set.

Model	Accuracy (%)	Recall (%)	Precision (%)	F1 Score (%)	Params (M)
ResNet50	95.59	95.70	95.45	95.57	23.51
ResNeXt50	95.69	95.25	96.07	95.65	22.98
ConvNext-t	94.55	94.20	94.83	94.51	49.46
DenseNet201	94.88	95.34	94.44	94.89	18.10

**Table 3:** Recognition results of different classification models.

Model	Backbone	Accuracy (%)	F1 Score (%)	Params (M)	Speed (ms)
C3D + Linear Classifier	C3D	88.92	88.82	78.41	1264
CNN + LSTM	ResNet50	91.52	91.49	24.30	29
TSN	ResNet50	91.62	91.56	23.52	635
R2plus1D	3D ResNet34	92.13	92.16	63.76	1104
I3D	3D ResNet50	93.32	93.29	28.04	116
ResNeXt50-T	ResNeXt50	95.69	95.65	22.98	29

## Data Availability

The dataset will be published at https://github.com/jhygithub123/pig-aggressive-behavior-recognition-dataset (accessed on 27 May 2023) in the future.
